# Prevalence of nonalcoholic fatty liver disease in rheumatoid arthritis: An updated systematic review and meta-analysis

**DOI:** 10.1097/MD.0000000000043641

**Published:** 2025-08-08

**Authors:** Abinash Mahapatro, Maryam Jafari, Herby Jeanty, Satabdi Sahu, Fatemeh Eslampanah, Mohammad Amouzadeh-Lichahi, Mohit Mirchandani, Nakka Raghuma, Dhruvan Patel, Elan Mohanty, Kishan Patel, Reyhaneh Pasandipour, Seyyed Mohammad Hashemi, Ehsan Amini-Salehi

**Affiliations:** a Hi-Tech Medical College and Hospital, Rourkela, Odisha, India; b Student Research Committee, School of Medicine, Anzali International Campus, Guilan University of Medical Sciences, Rasht, Iran; c The Brooklyn Hospital Center, Brooklyn, NY; d MKCG Medical College and Hospital, Berhampur, Ganjam, Odisha, India; e Student Research Committee, Kermanshah University of Medical Sciences, Kermanshah, Iran; f Montefiore Medical Center Wakefield Campus, Bronx, NY; g GSL Medical College and General Hospital, Rajamahendravaram, Andhra Pradesh, India; h Drexel University College of Medicine, Philadelphia, PA; i Mary Medical Center Apple Valley, Apple Valley, CA; j Department of Internal Medicine, Riverside Community Hospital, Riverside, CA; k Cardiovascular Research Center, Hormozgan University of Medical Sciences, Bandar Abbas, Iran; l Gastrointestinal and Liver Diseases Research Center, Guilan University of, Medical Sciences, Rasht, Iran.

**Keywords:** epidemiology, meta-analysis, nonalcoholic fatty liver disease, nonalcoholic steatohepatitis, prevalence, rheumatoid arthritis

## Abstract

**Background:**

Rheumatoid arthritis (RA) is a chronic autoimmune disorder associated with various comorbidities, including nonalcoholic fatty liver disease (NAFLD). The prevalence of NAFLD in RA patients remains inconsistently reported, necessitating a comprehensive meta-analysis to estimate its true prevalence. This study aimed to assess the prevalence of NAFLD in patients with RA by synthesizing data from multiple studies.

**Methods:**

A systematic search was performed across PubMed, Scopus, and Web of Science for studies published up to January 2025. Eligible studies that reported on the prevalence of NAFLD in RA patients were included. A random-effects model was applied, and sensitivity analyses were conducted to test the robustness of the findings. Statistical analyses were performed using Comprehensive Meta-Analysis Software (CMA) version 4

**Results:**

The pooled prevalence of NAFLD/nonalcoholic steatohepatitis (NASH) in RA patients was found to be 22.8% (95% CI = 7.8%–50.8%) with substantial heterogeneity (*I*² = 99.52%). The prediction interval for the prevalence of NAFLD/NASH ranged from 1% to 99%. Subgroup analysis showed varying prevalence rates based on diagnostic modalities: 33.3% for FibroScan, 43.9% for liver biopsy, and 30.5% for ultrasonography.

**Conclusion:**

The prevalence of NAFLD is remarkable in RA patients, with varying rates depending on diagnostic methods. The substantial heterogeneity observed across studies, reflected in the wide range of the prediction interval, underscores the need for further studies to provide a more precise estimate. Given the notable prevalence of NAFLD/NASH in RA patients, clinicians are suggested to consider this issue by implementing regular liver screening as a valuable part of RA patient care. More studies are essential to offer a clearer and more consistent understanding of NAFLD/NASH in this population.

## 1. Introduction

Rheumatoid arthritis (RA) is a chronic autoimmune disorder primarily affecting the joints, leading to inflammation, pain, and long-term disability.^[1,2]^ It has been associated with various comorbid conditions, including cardiovascular disease, diabetes, and metabolic syndrome.^[3–6]^ One such condition gaining attention in RA patients is NAFLD, which is the most common chronic liver disorder globally.^[7–10]^ NAFLD encompasses a spectrum of liver abnormalities, ranging from simple steatosis to NASH, and may progress to cirrhosis or hepatocellular carcinoma.^[11,12]^

The relationship between RA and NAFLD remains a topic of growing research interest. Several factors contribute to this association, including the chronic inflammatory state of RA, which may lead to metabolic disturbances like insulin resistance, obesity, and dyslipidemia.^[13–16]^ These metabolic abnormalities are known risk factors for NAFLD.^[17,18]^ Additionally, certain medications used in RA treatment, such as methotrexate (MTX) and corticosteroids, may influence liver health, either exacerbating or mitigating the risk of NAFLD.^[19–21]^

The prevalence of NAFLD in patients with RA is an important clinical concern due to the potential for liver complications, which may worsen overall health outcomes. Despite emerging evidence, the exact prevalence of NAFLD in this patient population remains inconsistent across studies, largely due to variations in diagnostic methods, sample sizes, and patient demographics.^[22–24]^ Given the inconsistencies in prevalence estimates, there is a need for a meta-analysis to more accurately assess the overall prevalence of NAFLD in RA patients. This meta-analysis aims to comprehensively assess the prevalence of NAFLD in individuals with RA. By aggregating data from multiple studies, this research will provide a clearer picture of the prevalence of NAFLD in the RA population.

## 2. Methodology

This meta-analysis was carried out in accordance with the guidelines set out by the Preferred Reporting Items for Systematic Reviews and Meta-Analyses (PRISMA).^[25]^ The study protocol was registered with the International Prospective Register of Systematic Reviews (ID: CRD420251007404). The primary objective of this study was to estimate the prevalence of NAFLD in patients with RA by synthesizing data from various peer-reviewed studies.

### 2.1. Search strategy

A detailed and systematic search was conducted across several databases, including PubMed, Scopus, and Web of Science, covering studies published up to January 30, 2025. No restrictions on language were applied. The following search terms were used: “RA,” “RA,” “non-alcoholic fatty liver disease,” “NAFLD,” and “ nonalcoholic steatohepatitis.” Relevant studies were also identified by reviewing the reference lists of key publications. The full search strategy for each database is outlined in Table S1, Supplemental Digital Content, https://links.lww.com/MD/P563).

### 2.2. Study selection and eligibility criteria

Eligible studies for this meta-analysis were those that examined the prevalence of NAFLD in RA patients or reported the number of RA patients with and without NAFLD. We excluded case reports, review articles, editorials, and studies that did not provide sufficient data for comprehensive analysis.

Two independent reviewers first screened the titles and abstracts of all identified studies. Full-text reviews were then conducted to confirm eligibility. Discrepancies between the reviewers were resolved through discussion or consultation with a third reviewer to ensure agreement.

### 2.3. Quality assessment

Each study included in the meta-analysis was independently evaluated for quality by 2 reviewers using the Joanna Briggs Institute Critical Appraisal Checklist.^[26–28]^ Any disagreements in assessments were resolved by consulting a third reviewer to reach a consensus and guarantee reliable quality ratings.

### 2.4. Data extraction

Data were extracted independently from each study using a standardized data extraction form. The information collected included study details (author, year, country), demographic data (age, gender), diagnostic criteria for RA and NAFLD, sample size, and prevalence rates of NAFLD. In cases of missing or unclear data, the corresponding authors were contacted for further clarification or to obtain the necessary information.

### 2.5. Statistical analyses

All statistical analyses were performed using CMA (version 4.0) to combine the prevalence estimates. A random-effects model was applied to account for potential heterogeneity across studies. Prevalence rates with 95% confidence intervals were presented as percentages. The degree of heterogeneity between studies was assessed using *I*² statistics, with values exceeding 50% indicating substantial variability. Sensitivity analyses were performed to test the robustness of the findings by sequentially excluding individual studies to observe their impact on the overall prevalence estimate.

Publication bias was evaluated by visually inspecting funnel plots and conducting Egger and Begg tests, with a *P*-value of <.1 considered as evidence of significant bias. Meta-regression analyses were conducted to investigate potential factors contributing to the observed heterogeneity.

## 3. Results

### 3.1. Study selection

**Figure 1.**
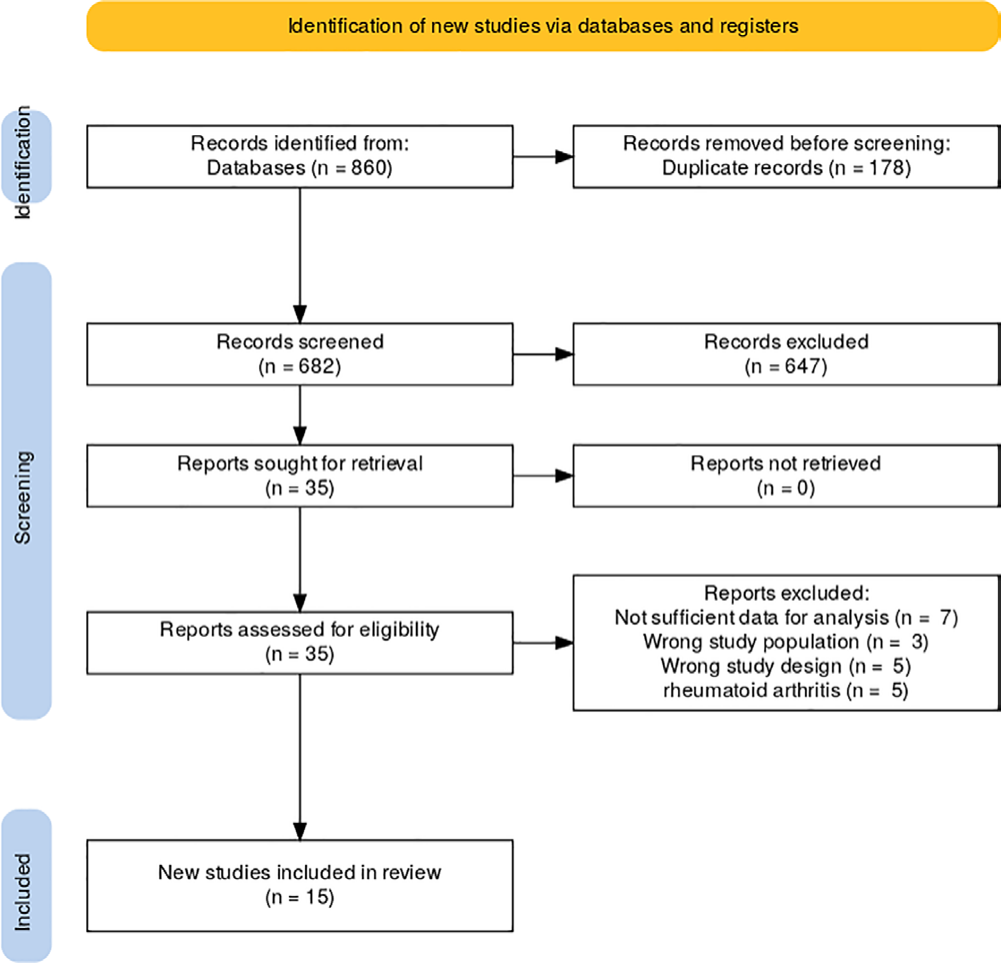
Study selection process.

The study selection process for the meta-analysis began with a total of 860 records identified from databases. After removing 178 duplicate records, 682 records were screened. During the screening process, 647 records were excluded for not meeting the inclusion criteria. Of the 35 remaining reports, 7 were excluded due to insufficient data for analysis, 3 were excluded for involving the wrong study population, 5 were excluded due to the wrong study design, and 5 were excluded because they did not focus on RA. In the end, a total of 15 new studies were included in the review (Fig. 1).

### 3.2. Study characteristics

This systematic review and meta-analysis synthesized data from 15 studies evaluating the prevalence of NAFLD in patients with RA. These studies were conducted across diverse geographical regions, including Spain,^[29]^ France,^[30]^ South Korea,^[22]^ Italy,^[24,31]^ Taiwan,^[7,32]^ Ukraine,^[33]^ Japan,^[34,35]^ the USA,^[36]^ Malaysia,^[37]^ Pakistan,^[23]^ India,^[38]^ and China,^[39]^ spanning publication years from 2014 to 2024 (Table 1).

**Table 1:** Key characteristics of included studies.

No.	Authors	Year	Country	Study population					NAFLD prevalence		Modality	RA criteria		
				Sample size	Mean age	MTX use (n)	Male	Female	No. patients	Prevalence (%)			Subgroup	Risk of bias
1	Castiella^[29]^	2023	Spain	59	61.52	59	16	43	17	28.8%	Ultrasound	NA	Steatosis	Low
2	Cervoni^[30]^	2020	France	40	58	25	14	26	19	47.5%	Ultrasound	NA	Steatosis	Low
				40	58	25	14	26	5	12.5%	NA	NA	NAFLD	Low
				40	58	25	14	26	15	37.5%	NA	NA	NAFLD	Low
				30	NA		NA	NA	10	33.3%	Fibroscan > F2	NA	NAFLD	Low
3	Choi^[22]^	2021	South Korean	437	NA	NA	NA	NA	92	21%	Ultrasound	ACR 1987/ ELAR 2009	NAFLD	Low
4	Erre^[31]^	2021	Italy	223	61	NA	62	161	188	84.3%	Ultrasound	ACR/EULAR 2010	Steatosis	Low
5	Ho ^[7]^	2024	Taiwan	2281	52	NA	510	1771	50	2.1%	Ultrasound	NA	NAFLD	Low
6	Khimion^[33]^	2023	Ukraine	126	NA	NA	24	102	77	61.1%	Ultrasound	ARA 1987	NAFLD	Low
7	Meng^[32]^	2024	Taiwan	21 457	52	NA	5080	16 377	399	1.8%	Ultrasound	ICD-9-CM	NAFLD	Low
8	Mori^[34]^	2018	Japan	32	NA	32	NA	NA	7	21.8%	Liver biopsy	NA	NAFLD	Low
				32	NA	32	NA	NA	22	68.7%	Liver biopsy	NA	NASH	Low
9	Mori^[35]^	2020	Japan	24	NA	24	NA	NA	3	12.5%	Liver biopsy	NA	NAFLD	Low
				24	NA	24	NA	NA	19	79.1%	Liver biopsy	NA	NASH	Low
10	Ogdie^[36]^	2018	USA	54,251	NA	NA	16,273	37,978	171	0.3%	NA	NA	NAFLD	Low
11	Sakthiswary^[37]^	2014	Malaysia	112	NA	NA	NA	NA	46	41%	Ultrasound	NA	NAFLD	Low
12	Ursini^[24]^	2017	Italy	164	NA	67	35	129	41	25%	Ultrasound	ACR/EULAR 2010	NAFLD	Low
13	Wagan^[23]^	2020	Pakistan	192	NA	167	36	156	39	20.3%	Ultrasound	NA	NAFLD	Low
14	Yadav^[38]^	2019	India	`493	NA	NA	NA	NA	3	0.6%	NA	ACR	NAFLD	High
15	Zou^[39]^	2022	China	513	51.8	NA	111	402	105	20.4%	Ultrasound	2010 criteria	NAFLD	Low

ACR = American College of Rheumatology, ARA = American Rheumatism Association, EULAR = European Alliance of Associations for Rheumatology, ICD-9-CM = International Classification of Diseases, 9th Revision, Clinical Modification, MTX = methotrexate, NA = not available, NAFLD = nonalcoholic fatty liver disease, NASH = nonalcoholic steatohepatitis, RA = rheumatoid arthritis.

The study populations exhibited substantial variability, with sample sizes ranging from 24 participants^[35]^ to large-scale cohorts exceeding 54,000 individuals.^[36]^ The reported mean age ranged between 51.8 years^[39]^ and 61.5 years.^[29,31]^ MTX use was documented in several studies, while gender distribution reflected the higher prevalence of RA in female patients.

The prevalence of NAFLD among RA patients demonstrated notable heterogeneity, varying from 0.3% in large population-based datasets^[36]^ to 84.3% in smaller, highly selective cohorts.^[31]^ Ultrasound was the predominant imaging modality employed for NAFLD diagnosis, though a subset of studies utilized liver biopsy^[34,35]^ or FibroScan^[30]^ to assess hepatic involvement. Some studies further distinguished between simple steatosis and progressive forms of liver disease, such as NASH.^[34,35]^

Heterogeneity was also observed in the RA classification criteria, with studies adopting different diagnostic frameworks. Most studies demonstrated a low risk of bias, with only 1 study classified as high risk.^[38]^

### 3.3. Results of meta-analysis

#### 3.3.1. Prevalence of NAFLD/NASH in RA

**Figure 2.**
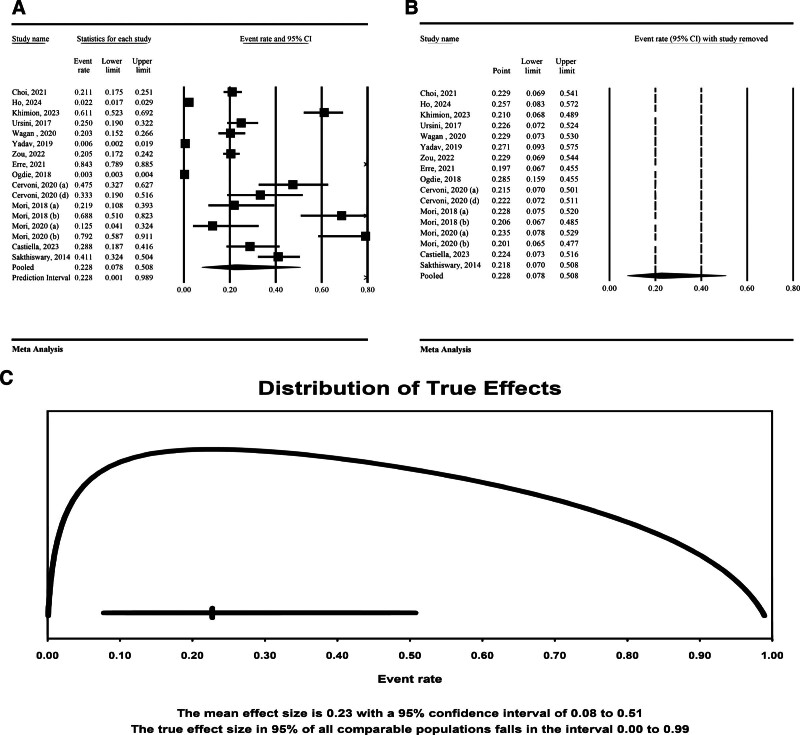
Prevalence of NAFLD in patients with RA. (A) Forest plot, (B) sensitivity analysis plot, (C) prediction interval analysis. NAFLD = nonalcoholic fatty liver disease, RA = rheumatoid arthritis.

The results of the study showed the prevalence of NAFLD/NASH in RA as 22.8% (95% CI = 7.8%–50.8%) (Fig. 2A). The results were accompanied by substantial heterogeneity (*I*² = 99.52%, *P* < .01). The sensitivity analysis showed that the pooled effect size remained stable after the omission of each individual study (Fig. 2B). Additionally, the prediction interval for the event rate was calculated to be between 0.01 and 0.99 (Fig. 2C).

#### 3.3.2. Prevalence of NAFLD/NASH in RA regarding diagnostic modality

**Figure 3.**
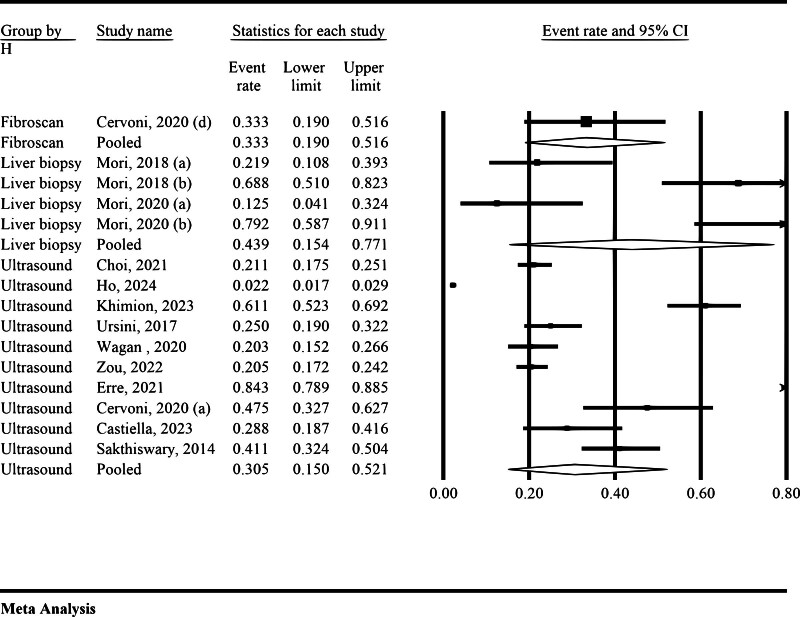
Prevalence of NAFLD in patients with RA regarding diagnostic modality. NAFLD = nonalcoholic fatty liver disease, RA = rheumatoid arthritis.

The results from Figure 3 indicate the prevalence NAFLD/NASH in patients with RA based on different diagnostic modalities. For Fibro scan, the pooled prevalence was 33.3% (95% CI = 19.0%–51.6%). The analysis revealed no significant heterogeneity (*I*² = 0.00%, and *P* = 1). For liver biopsy, the pooled prevalence was 43.9% (95% CI = 15.4%–77.1%), which showed substantial heterogeneity (*I*² = 90.01% and *P* < .01). For ultrasonography, the pooled prevalence was 30.5% (95% CI = 15.0%–52.1%), with a very high level of heterogeneity (*I*² = 98.7%, *P* < .01).

### 3.4. Results of meta-regression analysis

Meta-regression analyses were conducted to investigate the potential factors contributing to the prevalence of NAFLD/NASH among RA patients. The following variables were analyzed: age, body mass index (BMI), MTX use, liver enzymes (AST, ALT), triglyceride (TG), total cholesterol, comorbidities such as hypertension (HTN), and diabetes mellitus.

**Figure 4.**
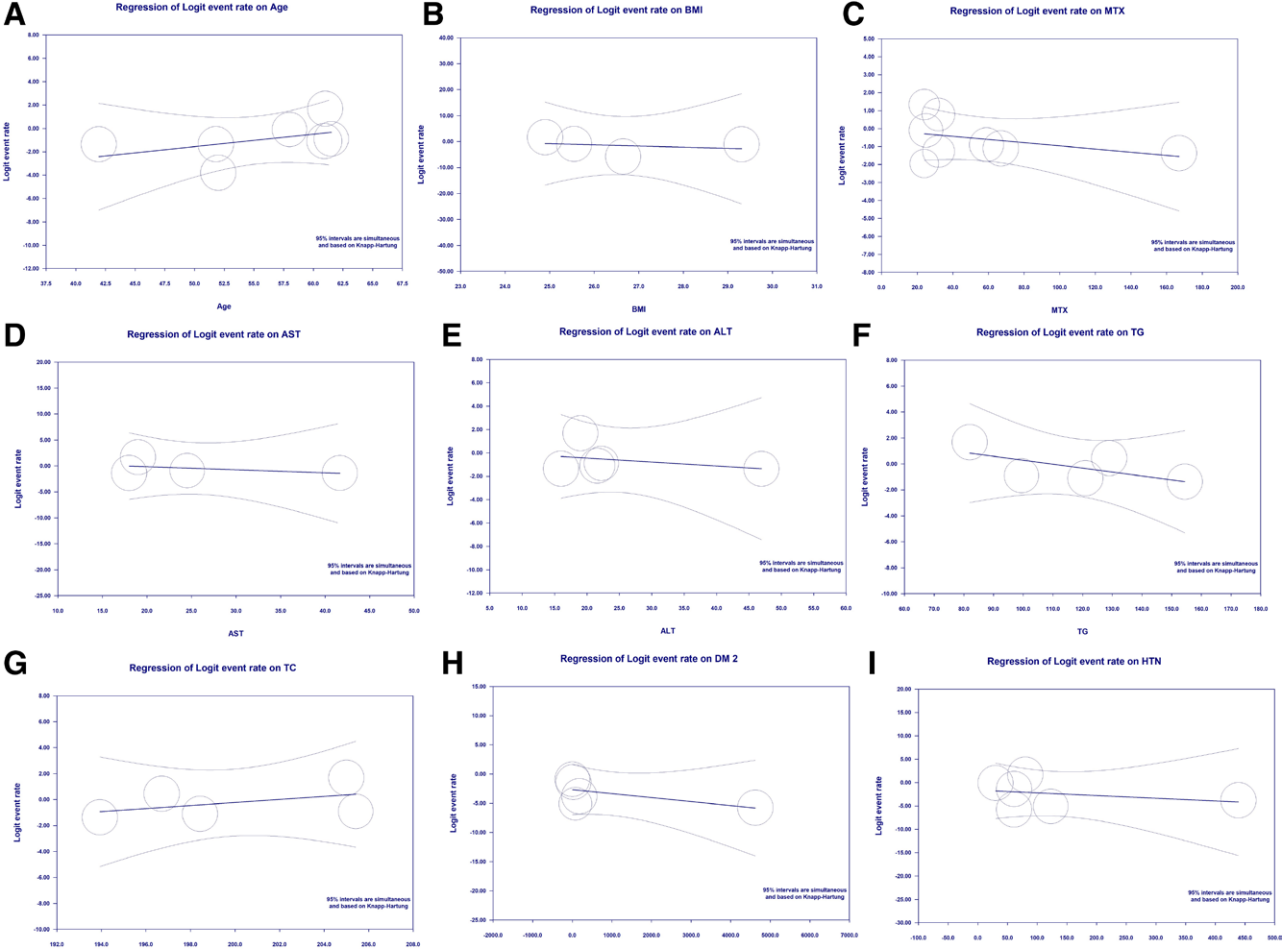
Results of meta-regression analysis of age (A), BMI (B), MTX use (C), AST (D), ALT (E), TG (F), TC (G), DM (H), HTN (I) on the NAFLD/NASH prevalence in RA. ALT = alanine aminotransferase, AST = aspartate aminotransferase, BMI = body mass index, DM = diabetes mellitus, HTN = hypertension, MTX = methotrexate, NAFLD = nonalcoholic fatty liver disease, NASH = nonalcoholic steatohepatitis, RA = rheumatoid arthritis, TC = total cholesterol, TG = triglycerides.

None of the factors, including age (coefficient: 0.10, *P* = .29), BMI (coefficient: −0.45, *P* = .71), MTX (coefficient: −0.07, *P* = .90), AST (coefficient: −0.05, *P* = .55), ALT (coefficient: −0.03, *P* = .59), TG (coefficient: −0.03, *P* = .22), total cholesterol (coefficient: 0.11, *P* = .43), DM2 (coefficient: 0.00, *P* = .23), or HTN (coefficient: −0.05, *P* = .41), had a significant effect on the prevalence of NAFLD/NASH in RA patients (Fig. 4).

## 4. Discussion

This meta-analysis provides a comprehensive assessment of the prevalence of NAFLD and NASH in patients with RA, revealing a pooled prevalence of 22.8% (95% CI = 7.8%–50.8%). However, the substantial heterogeneity observed (*I*² = 99.52%) indicates considerable variability across the included studies, likely reflecting differences in study populations, diagnostic methodologies, and underlying clinical characteristics. The wide prediction interval (1% to 99%) further underscores the heterogeneity, suggesting that the true prevalence of NAFLD in RA patients may vary substantially depending on specific population characteristics and diagnostic criteria. Notably, subgroup analyses demonstrated higher prevalence rates when liver biopsy (43.9%) and FibroScan (33.3%) were utilized, compared to ultrasonography (30.5%), which, despite its widespread use, may have lower sensitivity in detecting NAFLD. These findings highlight the considerable burden of NAFLD in RA patients and underscore the need for standardized diagnostic approaches and routine hepatic monitoring in this population to facilitate early detection and appropriate management strategies.

NAFLD/NASH represents a significant comorbidity in patients with RA, contributing to increased systemic inflammation, metabolic dysfunction, and cardiovascular risk. The coexistence of these conditions may create a bidirectional interplay, where chronic inflammation in RA exacerbates hepatic steatosis and fibrosis, while NAFLD further amplifies systemic inflammation, potentially worsening RA disease activity.^[7,40–42]^

A large population-based cohort study by Ho et al demonstrated that newly diagnosed RA patients had an increased risk of developing NAFLD within the first 4 years of disease onset, highlighting the role of systemic inflammation in hepatic dysfunction.^[7]^ Additionally, NAFLD in RA patients has been associated with higher cardiovascular morbidity, further emphasizing the need for early detection. Also, Recent study by Vassilopoulos et al found that the prevalence of steatotic liver disease in RA patients was significantly associated with increased arterial stiffness and cardiovascular events, independent of traditional metabolic risk factors.^[8]^ These findings suggest that NAFLD should be recognized not only as a liver disorder but also as a marker of systemic vascular risk in RA patients.

The presence of NAFLD/NASH may also impact treatment responses and medication tolerance,^[32,43,44]^ particularly in patients receiving MTX or corticosteroids.^[35,37]^ Methotrexate-induced hepatotoxicity has been extensively studied, and recent data suggest that RA patients with NAFLD may be at higher risk of hepatic fibrosis when treated with MTX, as reported by Mori et al.^[35]^ Given the clinical implications of NAFLD in RA, early identification, risk stratification, and integrated management approaches are essential to mitigate liver-related complications and improve long-term outcomes in this high-risk population.

The pathophysiological connection between RA and NAFLD is complex and driven by systemic inflammation, metabolic dysfunction, and medication-related effects. While RA is primarily an autoimmune joint disease, its chronic inflammatory state increases the risk of metabolic disorders, including NAFLD.^[41,45]^ Understanding the mechanisms underlying this relationship is essential for optimizing disease management and identifying potential therapeutic targets.

Chronic inflammation plays a fundamental role in linking RA to NAFLD. Persistent immune activation in RA leads to excessive production of pro-inflammatory cytokines such as TNF-α, IL-6, and IL-1β, which contribute to hepatic insulin resistance and lipid accumulation. TNF-α impairs insulin signaling in hepatocytes, increasing hepatic fat deposition and promoting fibrosis. Similarly, IL-6 activates hepatic stellate cells, exacerbating liver damage.^[46–50]^ The systemic inflammatory burden in RA fosters an environment conducive to NAFLD progression, suggesting that controlling inflammation may help mitigate liver-related complications in these patients.

Metabolic dysfunction is another key factor contributing to NAFLD in RA patients. Chronic inflammation disrupts normal metabolic processes, leading to insulin resistance, dyslipidemia, and increased visceral adiposity.^[51–53]^ These factors drive hepatic fat accumulation and impaired lipid metabolism, increasing NAFLD risk. Insulin resistance promotes free fatty acid influx into hepatocytes, while pro-inflammatory cytokines interfere with lipid oxidation.^[54]^ RA patients often exhibit a metabolic syndrome-like phenotype,^[55]^ further predisposing them to NAFLD.^[50]^ Addressing these metabolic disturbances through lifestyle interventions and targeted therapies may help reduce hepatic complications in RA.

Alterations in gut microbiota composition may serve as a link between RA and NAFLD.^[56–58]^ Patients with RA often exhibit gut dysbiosis, characterized by increased intestinal permeability and bacterial endotoxin translocation, which contribute to hepatic inflammation.^[59,60]^ Endotoxins such as lipopolysaccharides (LPS) activate hepatic immune responses, exacerbating liver injury.^[61,62]^ Additionally, gut-derived metabolites influence hepatic lipid metabolism and insulin sensitivity. Restoring microbial balance through dietary modifications, probiotics, and gut-targeted therapies could offer a promising approach to reducing NAFLD progression in RA patients. According to Bakinowska et al., gut dysbiosis contributes to inflammation and immune dysregulation in RA. Dietary interventions, such as high-fiber, omega-3-rich, and Mediterranean diets, may help restore microbial balance and reduce RA severity.^[63]^

Long-term use of RA medications may influence NAFLD risk. MTX and corticosteroids, commonly prescribed for RA, have been linked to hepatic steatosis and metabolic disturbances.^[7,37]^ While MTX can induce hepatic fibrosis in predisposed individuals, its exact contribution to NAFLD remains debated.^[19,34]^ Corticosteroids exacerbate insulin resistance and lipid accumulation, potentially worsening NAFLD.^[32]^ In contrast, biologic agents such as TNF inhibitors and IL-6 blockers may have protective effects by reducing systemic inflammation.^[48,64]^ A personalized approach to RA treatment, balancing disease control with liver health considerations, is necessary to minimize medication-related hepatic risks.

Genetic predisposition may contribute to NAFLD susceptibility in RA patients, with certain polymorphisms linked to hepatic fat accumulation and fibrosis. Shared genetic factors suggest a connection between RA and NAFLD, while epigenetic modifications may further influence liver disease risk.^[65–67]^ Identifying these factors could help stratify high-risk patients and guide targeted prevention.

The high prevalence of NAFLD in RA patients necessitates its consideration as a critical comorbidity in routine clinical practice. Given the increased risk of hepatic fibrosis and metabolic complications, systematic liver function assessment should be integrated into RA management.^[40,68]^ Noninvasive diagnostic tools, such as FibroScan and serum biomarkers, should be utilized to detect early-stage liver disease and guide clinical decision-making.^[69]^ Additionally, multidisciplinary collaboration between rheumatologists and hepatologists is essential for optimizing patient care. Lifestyle interventions, including dietary modifications, weight management, and physical activity, should be emphasized alongside pharmacologic strategies to minimize liver-related risks in RA patients.^[70–72]^

Previous meta-analysis by Zamani et al provided important insights into the prevalence of NAFLD among patients with RA, reporting a pooled prevalence of 35.3%.^[68]^ We acknowledge their systematic and valuable contribution, which helped establish a clear understanding of the relationship between NAFLD and RA.

Our findings showed the overall prevalence as 22.8%, which supports the conclusion that NAFLD is common among patients with RA. Although there are some numerical differences between the 2 analyses, both studies emphasize that NAFLD is an important health issue in this patient group and highlight the need for regular monitoring of liver health.

Importantly, our study extends the findings of Zamani et al through several key enhancements. Firstly, our literature search was updated and included more recent studies, covering publications up to January 2025, compared to August 2022 in their analysis. Additionally, our meta-analysis incorporated 15 original studies, compared to 9 studies in their work, increasing the reliability and generalizability of our results.

Secondly, we conducted detailed meta-regression analyses to identify potential factors influencing the variability of NAFLD prevalence. We examined multiple factors such as age, BMI, methotrexate use, liver enzymes, triglycerides, cholesterol, diabetes mellitus, and hypertension. Although these factors did not significantly explain the variability, our analysis provided additional insights into possible associations and laid the groundwork for future research.

Thirdly, our study included prediction interval analysis, providing a clearer view of the expected range of NAFLD prevalence in future studies. This statistical method helps clinicians and researchers better anticipate potential variations across different clinical settings and populations.

Lastly, our subgroup analyses according to diagnostic methods (FibroScan, liver biopsy, and ultrasound) highlighted how prevalence estimates can vary significantly based on the diagnostic approach used. This emphasizes the need for standardizing diagnostic criteria to achieve more consistent and comparable results.

## 5. Limitations and future directions

While this meta-analysis provides valuable insights into the prevalence of NAFLD/NASH in RA patients, several limitations should be considered. First, the studies included in this analysis employed different diagnostic modalities for detecting NAFLD, such as FibroScan, liver biopsy, and ultrasonography, which could contribute to the observed heterogeneity in prevalence estimates. The variability in diagnostic techniques, along with differences in study populations, sample sizes, and methodologies, makes it challenging to generalize the findings universally.

Additionally, factors such as medication use (e.g., methotrexate and corticosteroids), lifestyle habits, and comorbidities may have influenced the development and progression of NAFLD in this population, yet meta-regression analysis did not identify any significant associations. This suggests that more refined studies are needed to evaluate these potential contributors in greater detail.

Moreover, many of the studies included were cross-sectional in nature, which limits the ability to draw conclusions about causal relationships. Longitudinal studies would be valuable in understanding the progression of NAFLD/NASH in RA patients and identifying potential risk factors.

Future research should focus on standardizing diagnostic methods for NAFLD in RA patients and exploring the impact of various treatments on liver health. Investigating the molecular mechanisms linking RA and NAFLD, as well as conducting well-designed prospective studies, will help refine our understanding of this association. Additionally, larger, multi-center studies that examine diverse RA populations are necessary to provide more accurate prevalence estimates and help establish clinical guidelines for screening and management.

## 6. Conclusion

This study demonstrates that NAFLD/ NASH is prevalent among patients with RA, with a notable variation depending on the diagnostic method used. Despite significant heterogeneity in the findings, it is evident that NAFLD/NASH is an important comorbidity in this population. The wide range of the prediction interval reflects the need for further research to refine our understanding of the true prevalence and contributing factors. Clinicians should be aware of the substantial risk of NAFLD in RA patients and consider incorporating routine liver screening as part of comprehensive patient care. Given the limitations of the current data and the lack of significant predictors, it is clear that more targeted studies are required to better define the relationship between RA and NAFLD/NASH. These future studies should focus on standardizing diagnostic methods and exploring the impact of various treatment regimens on liver health to enhance clinical management and improve patient outcomes.

## Author contributions

**Conceptualization:** Abinash Mahapatro, Herby Jeanty, Elan Mohanty, Ehsan Amini-Salehi.

**Data curation:** Maryam Jafari, Herby Jeanty, Mohammad Amouzadeh-Lichahi, Ehsan Amini-Salehi.

**Formal analysis:** Dhruvan Patel, Ehsan Amini-Salehi.

**Investigation:** Abinash Mahapatro, Maryam Jafari, Satabdi Sahu, Kishan Patel, Ehsan Amini-Salehi.

**Methodology:** Satabdi Sahu, Mohit Mirchandani.

**Project administration:** Maryam Jafari, Nakka Raghuma, Kishan Patel.

**Resources:** Herby Jeanty, Nakka Raghuma, Elan Mohanty, Kishan Patel, Reyhaneh Pasandipour.

**Software:** Elan Mohanty.

**Supervision:** Ehsan Amini-Salehi.

**Validation:** Maryam Jafari, Ehsan Amini-Salehi.

**Visualization:** Mohammad Amouzadeh-Lichahi, Seyyed Mohammad Hashemi.

**Writing – original draft:** Abinash Mahapatro, Satabdi Sahu, Fatemeh Eslampanah, Mohit Mirchandani, Dhruvan Patel, Seyyed Mohammad Hashemi.

**Writing – review & editing:** Abinash Mahapatro, Satabdi Sahu, Fatemeh Eslampanah, Mohit Mirchandani, Dhruvan Patel, Ehsan Amini-Salehi.

## Supplementary Material


