# Supplementary material for: Prevalence of nonalcoholic fatty liver disease in rheumatoid arthritis: An updated systematic review and meta-analysis
Source: Medicine (Baltimore). 2025 Aug 8;104(32):e43641. doi: 10.1097/MD.0000000000043641 (PMC12338258; doi:10.1097/MD.0000000000043641)
Supplement: Supplementary file 1 [file medi-104-e43641-s001.docx]

**Table S1**: Search strategy of included studies

| Data base | Search formula |
| --- | --- |
| PubMed | (("Arthritis, Rheumatoid"[Mesh]) OR (Rheumatoid Arthritis[Title/Abstract])) AND ((((((("Non-alcoholic Fatty Liver Disease"[Mesh]) OR (NAFLD[Title/Abstract])) OR ("MASLD"[Title/Abstract])) OR ("MAFLD"[Title/Abstract])) OR ("Fatty Liver"[Title/Abstract])) OR ("Hepatic Steatosis"[Title/Abstract])) OR ("Metabolic Dysfunction-Associated Steatotic Liver Disease"[Title/Abstract])) |
| Scopus | TITLE-ABS-KEY ( "Rheumatoid Arthritis" OR "Arthritis, Rheumatoid" ) AND TITLE-ABS-KEY ( "Non alcoholic Fatty Liver Disease" OR "NAFLD" OR "Nonalcoholic Fatty Liver Disease" OR "Fatty Liver, Nonalcoholic" OR "Fatty Livers, Nonalcoholic" OR "Liver, Nonalcoholic Fatty" OR "Livers, Nonalcoholic Fatty" OR "Nonalcoholic Fatty Liver" OR "Nonalcoholic Fatty Livers" OR "Nonalcoholic Steatohepatitis" OR "Nonalcoholic Steatohepatitides" OR "Steatohepatitides, Nonalcoholic" OR "Steatohepatitis, Nonalcoholic" OR "Metabolic Dysfunction-associated Fatty Liver Disease" OR "MASLD" OR "MAFLD" OR " Metabolic Dysfunction-associated Fatty Liver Disease" ) |
| Web of Science | (("Rheumatoid Arthritis" OR "Arthritis, Rheumatoid") and ("Non alcoholic Fatty Liver Disease" OR "NAFLD" OR "Nonalcoholic Fatty Liver Disease" OR "Fatty Liver, Nonalcoholic" OR "Fatty Livers, Nonalcoholic" OR "Liver, Nonalcoholic Fatty" OR "Livers, Nonalcoholic Fatty" OR "Nonalcoholic Fatty Liver" OR "Nonalcoholic Fatty Livers" OR "Nonalcoholic Steatohepatitis" OR "Nonalcoholic Steatohepatitides" OR "Steatohepatitides, Nonalcoholic" OR "Steatohepatitis, Nonalcoholic" OR "Metabolic Dysfunction-associated Fatty Liver Disease" OR "MASLD" OR"MAFLD" OR " Metabolic Dysfunction-associated Fatty Liver Disease")) |
